# Platelet Lymphocyte Ratio and Mean Platelet Volume as Predictive Biomarkers for Spontaneous Passage of Distal Ureteric Stones in Patients Undergoing Medical Expulsive Therapy

**DOI:** 10.7759/cureus.96015

**Published:** 2025-11-03

**Authors:** Santosh Patil, Siddanagouda Patil, Vinay S Kundargi, H M Dhruva, Ruchitha Vinayak, Gulshan Kumar, Kiran K Negi

**Affiliations:** 1 Urology, Shri BM Patil Medical College, BLDE (Deemed to be University), Vijayapura, IND; 2 Anaesthesiology, LH Bidri Hospital, Bijapur, IND

**Keywords:** distal ureteric stones, mean platelet volume, medical expulsive therapy, platelet-lymphocyte ratio, urolithiasis

## Abstract

Introduction: Urolithiasis is a prevalent clinical condition associated with considerable morbidity. Distal ureteric stones are frequently managed with medical expulsive therapy (MET); however, predicting spontaneous stone passage remains a clinical challenge. Inflammatory markers such as platelet-lymphocyte ratio (PLR) and mean platelet volume (MPV) may serve as useful indicators for assessing the likelihood of MET success. This study aims to evaluate the predictive value of PLR and MPV in determining the success of MET for distal ureteral stones.

Materials and Methods: This prospective observational study enrolled 121 patients with single distal ureteric stones (5-10 mm). All patients received tamsulosin 0.4 mg/day for two weeks. PLR and MPV were measured at baseline, one week, and two weeks. Spontaneous passage was confirmed by imaging at two weeks. Differences between stone passers (n=100) and non-passers (n=21) were analyzed. Logistic regression identified independent predictors of stone passage, and receiver operating characteristic (ROC) analysis determined diagnostic performance and optimal cut-offs.

Results: Stone passers had significantly higher MPV and lower PLR compared to non-passers at all time points (p<0.001). ROC analysis showed good predictive performance for PLR (AUC=0.84, cut-off 20.5) and MPV (AUC=0.87, cut-off 7.5 fL). Logistic regression identified stone size >7 mm (OR 0.42, p=0.03), PLR ≤ 20.5 (OR 3.62, p<0.001), and MPV >7.5 fL (OR 4.28, p<0.001) as independent predictors of spontaneous stone passage.

Conclusion: PLR and MPV are significant, easily measurable hematological markers that can predict spontaneous passage of distal ureteric stones, aiding clinical decision-making and potentially reducing unnecessary interventions.

## Introduction

Urolithiasis, or the formation of stones within the urinary tract, is a prevalent medical condition with significant global health implications [1]. Within the Indian population, around 12% are projected to develop urinary stones, with half of these cases potentially leading to impaired kidney function [2]. Ureteral stones, representing around 20% of all urolithiasis cases, are associated with considerable morbidity and healthcare costs [3]. The management of ureteral stones depends on factors such as stone size, location, and the presence of complications [4,5]. For small distal ureteral stones, medical expulsive therapy (MET) is commonly employed to facilitate spontaneous stone passage, offering a non-invasive alternative to surgical interventions [6,7].

Imaging plays a central role in the diagnosis and management of ureteral stones [8]. Non-contrast computed tomography (NCCT) is considered the gold standard due to its high sensitivity and accuracy [9]. For small distal stones, MET is often used as first-line therapy [10]. This approach typically includes pharmacological agents such as alpha-blockers (e.g., tamsulosin), which relax ureteral smooth muscle and promote stone expulsion [11]. Despite its advantages, the success of MET is variable and depends on multiple factors, including stone size, location, and patient-specific characteristics [12]. Spontaneous stone passage rates decreased with stone size: 0-5 mm was 89%, 5-7 mm was 49%, and ≥7 mm was 29% [13].

Recent evidence suggests that systemic inflammatory markers may provide additional predictive insight regarding stone passage [14]. Platelet lymphocyte ratio (PLR), the ratio of platelet count to lymphocyte count, serves as a marker of systemic inflammation and has been associated with various inflammatory conditions [15-17]. Similarly, mean platelet volume (MPV), which reflects the average size of circulating platelets, has been linked to platelet activation and inflammatory processes, potentially influencing stone formation and expulsion [15,18]. To date, only a few studies have evaluated the role of PLR in predicting spontaneous ureteral stone passage, with at least one study reporting non-significant results [12]. No studies have specifically assessed the utility of MPV in this context, highlighting a gap in the literature and the need for further investigation. Hence, this study aims to evaluate the predictive value of PLR and MPV in determining spontaneous stone passage during MET.

## Materials and methods

This was a prospective observational study conducted in the Department of Urology at Shri BM Patil Medical College, Hospital and Research Centre, Vijayapura, Karnataka, India, between June 2023 and December 2024. The study aimed to evaluate the role of PLR and MPV as predictive biomarkers of spontaneous passage of distal ureteric stones in patients undergoing MET. The study was approved by the Institutional Ethics Committee (IEC No. BLDE (DU)/IEC/999/2022-23), and written informed consent was obtained from all participants.

The minimum required sample size was calculated to detect a difference in PLR between stone passers (spontaneous stone passage, SSP) and non-passers. Using the formula:

n = \frac{2 \, (Z_{\alpha/2} + Z_{\beta})^2 \, \sigma^2}{\Delta^2}

where Zα/2=1.96 (for 95% confidence), Zβ=0.84 (for 80% power), σ = pooled standard deviation, and Δ = expected mean difference. Based on Aghaways et al. [16], PLR values were 11.47 ± 4.86 in the no-SSP group and 9.00 ± 2.30 in the SSP group. The pooled SD was calculated as 4.32, and the mean difference (Δ) was 2.47. Substituting these values yielded a minimum of 48 patients per group (total 96). To increase statistical power and account for potential loss to follow-up, a total of 121 patients were included in this study.

A total of 121 patients aged 18 years or above with a single distal ureteric stone, ranging in size from 5 to 10 mm, with a confirmed diagnosis on NCCT, were enrolled. Patients were considered eligible if they were clinically stable and suitable for MET. Exclusion criteria included severe hydronephrosis, bilateral ureteric stones, symptomatic urinary tract infection, fever from other causes, acute renal failure, use of immunosuppressive therapy, active malignancy, or refusal to consent. Patients lost to follow-up or requiring immediate intervention were excluded.

All patients received tamsulosin 0.4 mg orally once daily for two weeks, along with advice for adequate hydration. No additional expulsive medications were prescribed. Medication adherence was reinforced during each follow-up visit, and patients were asked to maintain a medication diary and bring empty strips for verification. Patients were monitored clinically and reviewed after one and two weeks to assess treatment response. Spontaneous passage of the stone was defined as the disappearance of the stone on follow-up imaging and relief of symptoms. Patients were categorized into two groups: stone passers (MET success) and stone non-passers (MET failure).

Baseline demographic and clinical data, including age, sex, stone size, and laterality, were recorded. Venous blood samples were obtained from the antecubital vein in the morning after an overnight fast, at baseline, one week, and two weeks. Samples were collected in ethylenediaminetetraacetic acid (EDTA) tubes and processed within an hour of collection using a standardized protocol to avoid platelet swelling. Complete blood count and inflammatory markers were analyzed using an automated haematology analyser (Sysmex XN-1000, Sysmex Corp., Kobe, Japan). PLR was calculated as the ratio of platelet count to lymphocyte count, while MPV was recorded directly from the analyzer output. To ensure consistency, all analyses were performed in the same laboratory by the same technician team.

PLR and MPV were initially analyzed as continuous variables. For logistic regression and ROC analysis, they were subsequently dichotomized based on optimal cut-off values derived from ROC curves (PLR ≤20.5, MPV >7.5 fL). Potential confounding variables, including age, sex, and stone size, were assessed and adjusted in multivariate regression models. Imaging was performed for all patients at two weeks using both a kidney, ureter, and bladder (KUB) X-ray and ultrasonography (USG KUB) to document stone clearance or persistence. Ultrasound was conducted using a high-resolution 3.5-5 MHz convex probe, and all scans were performed by a single experienced radiologist blinded to laboratory data to minimize bias.

Statistical analysis was carried out using IBM SPSS Statistics v. 27 (IBM Corp., Armonk, NY). Continuous variables were expressed as mean ± standard deviation (SD) and compared using independent t-tests. Categorical variables were analyzed with chi-square tests. Logistic regression was used to identify independent predictors of stone passage, and receiver operating characteristic (ROC) curve analysis was applied to determine optimal cut-off values for PLR and MPV. A p-value <0.05 was considered statistically significant.

## Results

The study population comprised 121 patients with a single distal ureteric stone, of whom 100 (82.6%) achieved spontaneous stone passage (stone passers) and 21 (17.4%) did not (non-passers). The mean age of participants was 42.8 ± 12.1 years, with no significant difference between stone passers (41.9 ± 11.9) and non-passers (46.2 ± 12.5) (p = 0.21). The majority of patients were male (65.3%), and stone laterality was almost evenly distributed between right (n=63; 52.1%) and left (n=58; 47.9%) sides. Stone size was significantly larger in non-passers (7.8 ± 1.5 mm) compared to passers (6.9 ± 1.3 mm; p = 0.04), suggesting a potential influence of stone dimension on MET outcome (Table 1).

**Table 1:** Baseline Characteristics of the Study Population (n = 121) Data are presented as mean ± standard deviation (SD) for continuous variables and number (percentage) for categorical variables. Comparison between stone passers (n = 100) and non-passers (n = 21) was performed using the independent sample t-test for continuous variables and chi-square (χ²) test for categorical variables. SD = standard deviation; χ² = chi-square test; mm = millimeters

Variable		Total (n=121)	Stone Passers (n=100)	Non-Passers (n=21)	Test Statistic	p-value
Age (years)	Mean ± SD	42.8 ± 12.1	41.9 ± 11.9	46.2 ± 12.5	t = 1.26	0.21
Gender	Male	79 (65.3%)	65 (65.0%)	14 (66.7%)	χ² = 0.021	0.88
	Female	42 (34.7%)	35 (35.0%)	7 (33.3%)		
Stone size (mm)	Mean ± SD	7.1 ± 1.4	6.9 ± 1.3	7.8 ± 1.5	t = 2.08	0.04
Laterality	Right	63 (52.1%)	53 (53.0%)	10 (47.6%)	χ² = 0.20	0.65
	Left	58 (47.9%)	47 (47.0%)	11 (52.4%)		

PLR and MPV demonstrated clear differences between stone passers and non-passers over the course of treatment. At baseline, PLR was higher in non-passers (23.4 ± 7.1) than in passers (18.9 ± 5.9; p = 0.02) and remained consistently elevated in non-passers at one week (25.6 ± 6.9 vs. 17.8 ± 5.2; p < 0.001) and two weeks (29.1 ± 6.7 vs. 17.2 ± 4.8; p < 0.001). MPV values were conversely higher in stone passers at all time points, with a baseline of 8.6 ± 1.1 versus 7.2 ± 1.0 in non-passers (p < 0.001), and maintained a similar trend at one week (8.8 ± 1.0 vs. 7.0 ± 1.2; p < 0.001) and two weeks (8.7 ± 1.0 vs. 6.9 ± 1.2; p < 0.001), indicating a potential role of these biomarkers in predicting stone passage (Table 2).

**Table 2:** PLR and MPV Values at Baseline, One Week, and Two Weeks Data are presented as mean ± SD. Independent sample t-test was applied to compare groups at each time point. PLR = platelet-lymphocyte ratio; MPV = mean platelet volume; SD = standard deviation; fL = femtoliters

Parameter	Time Point	Stone Passers (n=100, mean ± SD)	Non-Passers (n=21, mean ± SD)	Test Statistic	p-value
PLR	Baseline	18.9 ± 5.9	23.4 ± 7.1	t = 2.38	0.02
	1 week	17.8 ± 5.2	25.6 ± 6.9	t = 5.06	<0.001
	2 weeks	17.2 ± 4.8	29.1 ± 6.7	t = 7.61	<0.001
MPV (fL)	Baseline	8.6 ± 1.1	7.2 ± 1.0	t = 4.55	<0.001
	1 week	8.8 ± 1.0	7.0 ± 1.2	t = 6.39	<0.001
	2 weeks	8.7 ± 1.0	6.9 ± 1.2	t = 6.29	<0.001

ROC curve analysis demonstrated that both PLR and MPV at two weeks had good predictive performance for spontaneous stone passage. PLR had an area under the curve (AUC) of 0.84 (95% CI: 0.75-0.92) with an optimal cutoff of 20.5, yielding a sensitivity of 92.0%, specificity of 76.2%, positive predictive value (PPV) of 94.8%, and negative predictive value (NPV) of 68.0%. MPV showed an AUC of 0.87 (95% CI: 0.79-0.95) with a cutoff of 7.5 fL, achieving 90.0% sensitivity, 81.0% specificity, 96.4% PPV, and 62.9% NPV. These results highlight the strong diagnostic potential of PLR and MPV for predicting MET success (Table 3).

**Table 3:** Diagnostic Accuracy of PLR and MPV at Two Weeks ROC curve analysis was used to derive the optimal cut-off values. ROC = receiver operating characteristic; AUC = area under the curve; CI = confidence interval; PLR = platelet-lymphocyte ratio; MPV = mean platelet volume; fL = femtoliters; PPV = positive predictive value; NPV = negative predictive value

Parameter	AUC (95% CI)	Optimal Cut-off	Sensitivity (%)	Specificity (%)	PPV (%)	NPV (%)
PLR	0.84 (0.75–0.92)	20.5	92.0	76.2	94.8	68.0
MPV (fL)	0.87 (0.79–0.95)	7.5	90.0	81.0	96.4	62.9

Logistic regression analysis identified stone size, PLR, and MPV at two weeks as significant independent predictors of stone passage. Stones larger than 7 mm were associated with lower odds of passage (OR 0.42; 95% CI: 0.18-0.87; p = 0.03). In contrast, a PLR lesser than 20.5 was associated with higher odds of stone passage (OR 3.62; 95% CI: 1.88-6.75; p < 0.001), as was an MPV greater than 7.5 fL (OR 4.28; 95% CI: 2.01-8.14; p < 0.001), confirming the predictive utility of these biomarkers in MET-treated patients (Table 4).

**Table 4:** Logistic Regression Analysis of Predictors of Stone Passage Odds ratios (OR) with 95% CI and p-values are presented. OR = odds ratio; CI = confidence interval; PLR = platelet-lymphocyte ratio; MPV = mean platelet volume; fL = femtoliters; MET = medical expulsive therapy; mm = millimeters

Variable	Odds Ratio (OR)	95% CI	p-value
Stone size (>7 mm)	0.42	0.18–0.87	0.03
PLR at 2 weeks (≤20.5)	3.62	1.88–6.75	<0.001
MPV at 2 weeks (>7.5 fL)	4.28	2.01–8.14	<0.001

## Discussion

Our study demonstrated that both the PLR and MPV are significant predictors of spontaneous passage of distal ureteral stones in patients undergoing MET. Notably, PLR was inversely associated with stone passage, with higher values correlating with lower odds of spontaneous expulsion. Conversely, MPV exhibited a positive correlation, where higher values were associated with an increased likelihood of stone passage. These findings align with previous research suggesting that systemic inflammation, as indicated by PLR, may impede stone passage, while increased platelet activity, reflected by MPV, could facilitate it. For instance, a study by Abou Heidar et al. found that elevated PLR was associated with reduced spontaneous passage rates [17].

The spontaneous passage rate observed in our study was 82.6%, with stone size being a significant factor. Patients with stones larger than 7 mm had a lower chance of spontaneous passage, consistent with findings from previous studies. For example, a study by Jendeberg et al. (2017) reported that stones larger than 6.5 mm had a spontaneous passage rate of only 9% [19]. Similarly, a meta-analysis by Park et al. (2021) indicated that stones larger than 5 mm had a spontaneous passage rate of 212 (35%), while those smaller than 5 mm had a rate of 394 (65%) [20].

Our study observed that MPV values were higher in stone passers compared to non-passers at all time points, suggesting a potential role of platelet activity in facilitating stone passage. To date, no studies have specifically evaluated the relationship between MPV and spontaneous stone passage, making our findings novel in this context. In contrast, PLR has been more extensively studied: Abou Haidar et al. reported a significant association between elevated PLR and reduced spontaneous passage rates, whereas Hasan et al. found no significant association, highlighting the variability in PLR’s predictive value across different populations [12,17].

The diagnostic performance of PLR and MPV in predicting spontaneous stone passage was supported by our ROC curve analysis, with both biomarkers demonstrating good predictive ability and MPV showing a higher AUC compared to PLR. This suggests that MPV may be a more reliable predictor of spontaneous passage. While no previous studies have specifically evaluated the diagnostic accuracy of PLR or MPV for ureteral stone passage, other studies have identified alternative variables, such as stone size, location, and CRP levels, as significant predictors of spontaneous stone expulsion [12,21,22].

This study has several limitations that should be considered when interpreting the results. The relatively small sample size and single-center design may limit the generalizability of the findings, and the short two-week follow-up could underestimate delayed or recurrent stone passage. Although medication adherence was encouraged and monitored, variations in compliance and unmeasured comorbidities such as diabetes or hypertension may have influenced outcomes. The imaging methods used (USG and X-ray KUB) have lower diagnostic accuracy compared with CT, which may have introduced misclassification bias. The study also controlled for a limited number of confounders, so residual confounding cannot be excluded. Therefore, multicenter studies with larger cohorts, longer follow-up periods, and external validation are recommended to confirm these findings and better establish the clinical applicability of PLR and MPV as predictive biomarkers for spontaneous ureteric stone passage.

## Conclusions

The study underscores the utility of simple hematological markers in anticipating the outcome of MET for distal ureteral stones. MPV and PLR reflect the underlying inflammatory and physiological processes influencing stone passage. Incorporating these routinely available parameters into clinical assessment may enhance early prediction of treatment success, support individualized management decisions, and potentially reduce the need for unnecessary interventions.
